# *In Vivo* Evaluation of Safety and Toxicity of a *Lactobacillus jensenii* Producing Modified Cyanovirin-N in a Rhesus Macaque Vaginal Challenge Model

**DOI:** 10.1371/journal.pone.0078817

**Published:** 2013-11-12

**Authors:** Beda Brichacek, Laurel A. Lagenaur, Peter P. Lee, David Venzon, Dean H. Hamer

**Affiliations:** 1 Vaccine Branch, National Cancer Institute, National Institutes of Health, Bethesda, Maryland, United States of America; 2 Osel, Incorporated, Mountain View, California, United States of America; 3 Biostatistics and Data Management Sect., National Cancer Institute, National Institutes of Health, Rockville, Maryland, United States of America; Charité-University Medicine Berlin, Germany

## Abstract

Sexual transmission of human immunodeficiency virus type 1 (HIV-1) across the cervicovaginal mucosa in women is influenced by many factors including the microbiota and the presence of underlying inflammation. It is important that potential HIV preventative agents do not alter the mucosal environment in a way that enhances HIV acquisition. We examined the impact of a “live” microbicide on the vaginal mucosal environment in a rhesus macaque repeated vaginal simian-HIV (SHIV_SF162P3_) challenge model. The microbicide contained a human vaginal *Lactobacillus jensenii* expressing the HIV-1 entry inhibitor, modified Cyanovirin-N (mCV-N), and henceforth called LB-mCV-N. Macaques were colonized vaginally each week with LB-mCV-N and sampled six days after colonization for culturable bacteria, pH and cervical-vaginal cytokines during the duration of the six-week study. We show that macaques that retained the engineered LB-mCV-N strain in their vaginal microbiota, during SHIV challenge, had lower pH, when colonization levels were higher, and had no evidence of inflammatory cytokines. Indeed, Interleukin-13, a mediator of inflammation, was detected less often in LB-mCV-N colonized macaques than in controls and we found higher levels of Interleukin 1 receptor antagonist (IL-1RA) in LB-mCV-N colonized macaques during the SHIV challenge period. We noted an inverse correlation between levels of mucosal IL-1RA and peak plasma viral load, thus higher IL-1RA correlated with lower viral load in LB-mCV-N treated macaques. These data support the use of LB-mCV-N as a safe “live” microbicide and suggest that lactobacilli themselves may positively impact the mucosal environment.

## Introduction

The majority of human immunodeficiency virus type 1 (HIV-1) transmissions in women occur during heterosexual intercourse when the virus penetrates mucosal surfaces of the vagina and cervix to reach target cells [1]–[4]. HIV-1 acquisition is potentiated by genital tract inflammation, which is often the result of an asymptomatic sexually transmitted infection [5]–[9]. Likewise, changes in the vaginal microbiota, found in conditions such as bacterial vaginosis, characterized by increased vaginal pH and higher levels of pro-inflammatory cytokines in cervical-vaginal secretions, lead to a higher probability of HIV-1 transmission [9]–[15]. The successful CAPRISA-004 trial, which tested 1% tenofovir gel, found that genital inflammation undermined gel effectiveness; thus, women with sufficient drug levels but inflamed epithelium were less protected than women without inflammation [16]. Finally, inflammation at the time of HIV-1 infection was found to be predictive of higher plasma viral load set point in women [17].

Early prevention trials of non-specific microbicides, such as Nonoxynol-9 (N-9) and cellulose sulfate (CS), may have contributed to increased rates of infections among users due to inflammation and disruption of the innate mucosal defenses [18]–[20]. Recently, it was also shown that N-9 and CS alter the vaginal microbiota [21]. Several groups have described methods for evaluation of biomarkers associated with cervical/vaginal mucosal barrier function and inflammation [22]–[28]. Evaluation of these biomarkers in the presence of a microbicide can be used as a guide to screen potential candidates for safety prior to their introduction into clinical settings.

We previously examined the efficacy of a live microbicide, *Lactobacillus jensenii* 1153–1666 expressing the HIV-1 entry inhibitor, modified Cyanovirin-N, hence called LB-mCV-N, in a repeated SHIV_SF162P3_ challenge vaginal model, and demonstrated that colonized animals showed a 63% reduction in acquisition of the virus and 6-fold reduced peak viral load [29], see File S1. During the SHIV challenge experiment, we examined the impact of this live microbicide, LB-mCV-N, on the vaginal microbiota, vaginal pH and vaginal biomarkers present in rhesus macaques. In this paper we present the findings of these studies.

## Results

### Analysis of the Culturable Vaginal Microbiome during Challenge

**Figure 1:**
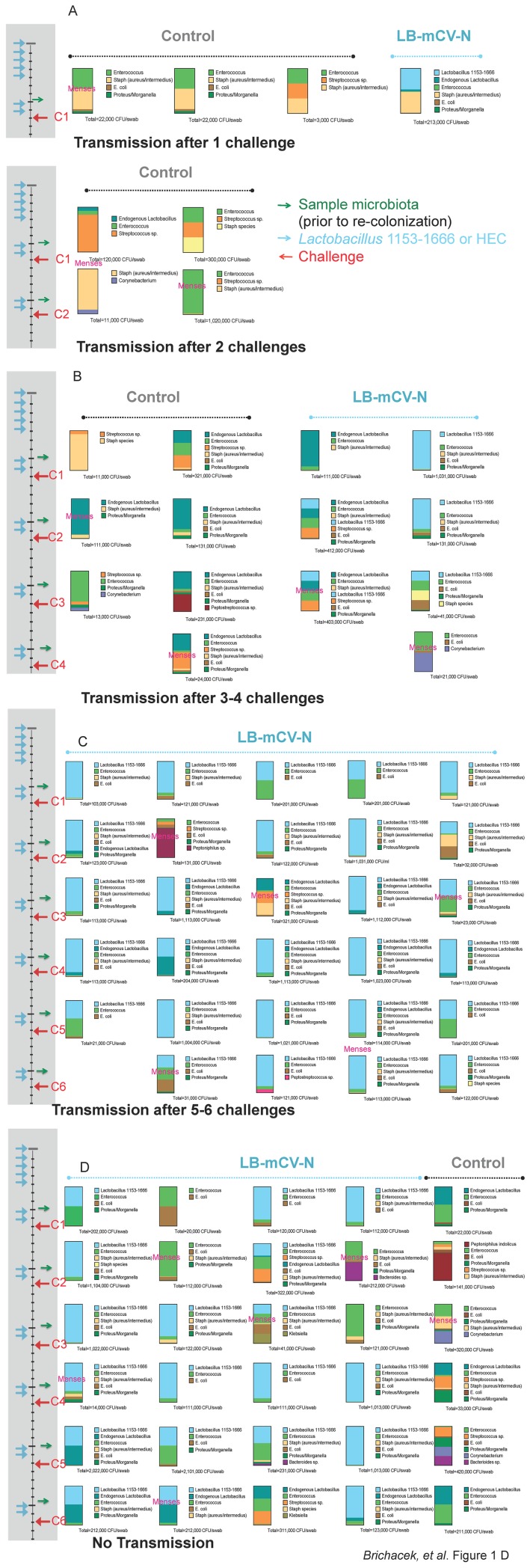
Culturable vaginal microbiota up to the time of transmission for mLB-CV-N colonized and control macaques. Each vertical column represents one macaque. Samples were taken two days prior to each challenge and cultured on standard and selective plating media. Bacteria were identified by Gram stain, API biochemical analysis and 16S rRNA sequence. Stacked bar graphs are used to show the relative percentage of the culturable vaginal microbiota within the macaque at the time of sampling and the number of colony forming units (CFU) isolated per swab, prior to re-colonization. The time frame for the six SHIV_SF162P3_ challenges (C1–6) is shown on the left; each challenge is represented as a red arrow, sampling is represented as a green arrow, re-colonization with LB-mCV-N or HEC treatment of macaques is represented as a light blue arrow. The first row of stacked bar graphs is pre-challenge. Macaques were arbitrarily divided into four groups, A) macaques that became infected at 1 or 2 challenges included five controls and one LB-mCV-N treated animal, B) macaques that became infected at 3–4 challenges included two controls and two LB-mCV-N treated, C) five macaques that became infected at 5–6 challenges were all treated with LB-mCV-N and D) macaques that resisted 6 challenges included four LB-mCV-N and one control. Menses that occurred on the day of sampling, is noted on the stacked bar graphs, menses that occurred during routine monitoring is noted between the stacked bar graphs. A Shannon Diversity Index was performed; no differences in bacterial diversity were noted in LB-mCV-N treated vs. the controls (p = 0.11 by Mann–Whitney-Wilcoxon test).

The vaginal microbiome is known to play an important role in the health of the lower genital tract**.** We analyzed the culturable vaginal microbiota of the experimental macaques, both those repeatedly colonized (n = 12) with LB-mCV-N and uncolonized (HEC treated) controls (n = 8) during the SHIV challenge experiment. We present the microbiota, as stacked bar graphs that represent the proportions of each bacterium and include the colony forming units (CFU) cultured from each swab (by a semi-quantitative method). Pre-challenge graphs are presented for all macaques and then subsequent microbiota each week until the macaque became infected. Animals were divided arbitrarily into those that became infected early, 1–2 challenges (Figure 1A), intermediate, 3–4 challenges (Figure 1B), late 5–6 challenges (Figure 1C) or those that remained uninfected (Figure 1D). Five control macaques and one LB-mCV-N macaque became infected early (Figure 1A). Menses is noted, when it occurred on the day of sampling (overlaying the stacked bar) or as part of daily routine observations (between bars). LB-mCV-N colonization was associated with longer time to acquisition of SHIV. Seven of eight controls were infected at early or intermediate challenges, while one control resisted infection. Conversely only three of 12 LB-mCV-N colonized macaques were infected at early or intermediate challenges, five were infected late and four resisted infection. *Lactobacillus, Streptococcus* and *Enterococcus* species, all lactic acid producing organisms were cultured from the macaque vagina. *Enterobacteriaceae,* hemolytic *Staphylococcus aureus/intermedius* and facultative anaerobic sp., including *Bacteroides sp*., *Peptostreptococcus sp*., and *Peptoniphilus sp.* were also isolated. Non-hemolytic *Staphylococcus*, *Micrococcus* and *Corynebacterium sp.* were occasionally isolated. The vaginal microbiome in the macaque is dynamic. A Shannon Diversity Index, a measure a total number of species in the community, was performed on the microbiology sample analysis. No differences in bacterial diversity were noted in the *Lactobacillus* treated vs. HEC placebo treated macaques (statistical significance p = 0.11 by the Mann-Whitney-Wilcoxon test). Thus addition of LB-mCV-N did not change the overall diversity of the vaginal microbiota.

### Examination of the Menstrual Cycle and Influences on Microbiome and SHIV Susceptibility

Menstruation may have an effect on the stability of the vaginal microbiome, including decreasing the numbers of *Lactobacillus* and other species, as we previously have reported [30]. We observed menses during our challenge experiment and found no correlation between menstruation and SHIV infection. Notably, menstruation on the day of microbiology sampling did appear to impact the stability of the vagina microbiota in macaques that were colonized with LB-mCV-N (Figure 1, menses is noted), with a shift toward facultative anaerobic species, *Staphylococcus* sp., *Enterococcus* sp. or *Enterobacteriaceae*. Late luteal phase, which occurs just prior to menstruation was reported to be associated with increased susceptibility to SHIV vaginal infection in pigtail macaques [31]. We found that among the LB-mCV-N colonized macaques with regular menstruation cycles, 19 challenges during the luteal phase resulted in 3 infections while 8 challenges during the follicular phase produced 1 infection. This difference was not statistically significant.

### Analysis of Vaginal pH Related to Levels of *Lactobacillus* Colonization

**Figure 2:**
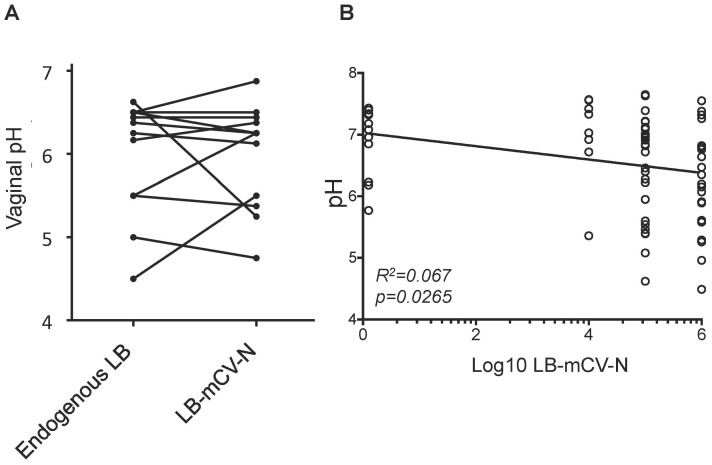
Influences of Lactobacillus on the vaginal pH of macaques. A) Vaginal pH was the same for macaques colonized with LB-mCVN or endogenous LB. Macaques colonized with endogenous *Lactobacillus* (LB) followed by colonization with LB-mCV-N had similar pH, no statistical different was noted in the vaginal pH with the different *Lactobacillus* species. B) Vaginal pH was measured and plotted against the levels of colonization with LB-mCV-N. Values on the far left represent times when macaques had no LB-mCV-N, and the other values are log levels of colony forming units (CFUs) of LB-mCV-N. Lower pH trended with LB-mCV-N. By simple linear regression, lower pH trended with LB-mCV-N (R^2^ = 0.067, slope p = 0.027). Macaques that were colonized with the highest levels of LB-mCV-N, at least 10^6^ colony forming units (CFU), were lower by an estimated 0.4 pH units from those with no colonization (p = 0.0058 by repeated measures analysis of variance).

Lactobacilli are known to lower vaginal pH in humans. To determine whether LB-mCV-N played a role in lowering vaginal pH in the macaque model, we first measured vaginal pH in macaques that were originally colonized with endogenous strains of *Lactobacillus* and then recolonized with LB-mCV-N strain and showed that both types of lactobacilli contribute similarly to vaginal pH (Figure 2A). Vaginal pH in macaques that were colonized with the highest levels of LB-mCV-N, at least 10^6^ colony forming units (CFU), were lower pH by an estimated 0.4 pH units from those with no colonization (p = 0.0058, Figure 2B). The trend across all the LB-mCV-N levels between no colonization and having at least 10^6^ CFU was neither significantly positive nor negative.

### Examination of Mucosal Biomarkers of Inflammation during Colonization and SHIV Challenge

Increases in the levels of proinflammatory or anti-inflammatory cytokines in the cervical-vaginal environment can enhance or reduce the susceptibility to HIV. To determine whether the LB-mCV-N microbicide altered biomarkers in the mucosal environment, we measured cytokines known for their effect on HIV transmission. We used a Luminex 23-plex assay to measure G-CSF, GM-CSF, IFN-γ, IL-1β, IL-1RA, IL-2, IL-4, IL-5, IL-6, IL-8, IL-10, IL12/23(p-40), IL-13, IL-15, IL-17, MCP-1, MIP-1β, MIP-1α, sCD40L, TGFα, TNFα, VEGF, and IL-18, in the CVLs of control and LB-mCV-N colonized macaques upon initial colonization and during the challenge experiment. Biomarkers were analyzed in an exploratory mode without correction for multiple comparisons but in consideration of independent results from previous studies.

During the period of initial colonization, we observed that IL-13, a mediator of allergic inflammation, was detectable in the CVL of 7/8 control macaques, but was only detectable in 3/12 CVLs from LB-mCV-N colonized macaques (p = 0.0198* Fishers t-test; data not shown). Thus it appears that colonization with LB-mCV-N suppressed the levels of the proinflammatory IL-13. No other cytokines were altered upon initial colonization (pre-challenge values).

**Figure 3:**
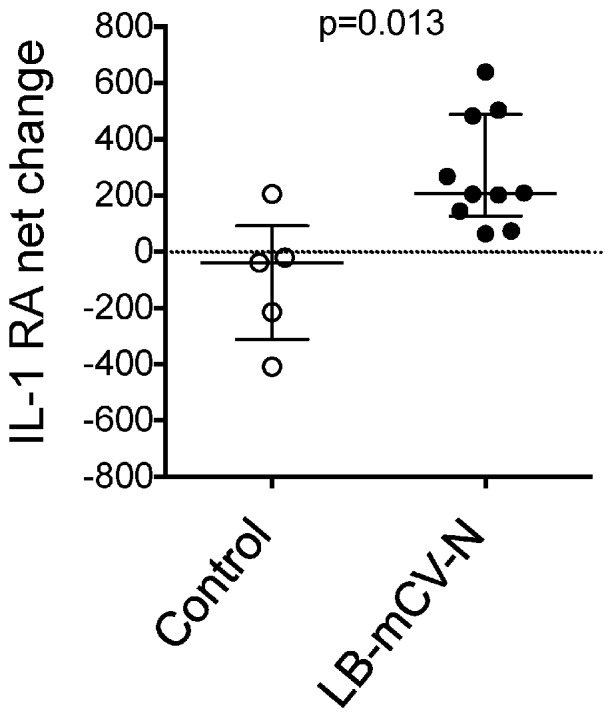
IL-1RA concentrations from cervical vaginal lavage (CVL) samples of control or LB-mCV-N colonized macaques during the SHIV challenge experiment. IL-1RA levels are reported as the net change in cytokine with the baseline (pre-challenge) values subtracted. IL-1RA was significantly higher in LB-mCV-N colonized macaques during challenge, p = 0.013. Note: SHIV transmission occurred at the first challenge in four animals (three controls and one LB-mCV-N), thus there were insufficient values available for baseline subtraction, and these animals were excluded from the analysis. One LB-mCV-N animal was missing a pre-challenge sample and was also excluded.

During the time period of the repeated vaginal SHIV challenge, we measured cytokines in the CVL’s of control and LB-mCV-N colonized macaques. We subtracted the pre-challenge cytokine background and looked for changes that occurred due to the interaction of the live microbicide on the epithelium in the presence of SHIV virus. In macaques that were dosed repeatedly with LB-mCV-N and challenged with SHIV, IL-1RA was found to be upregulated in CVLs compared to the levels in CVLs of controls (p = 0.013) (Figure 3). There were no statistical changes noted in any other proinflammatory biomarkers IL-1β, IL-6, TNFα, GM-CSF, IFN-γ, IL-8, IL12/23(p-40), IL-17, and IL-18, measured prior to and during the repeated challenges. Table 1 shows the minimum and maximum ranges and median levels of proinflammatory cytokines in the macaques. The table is divided to show the proinflammatory cytokine levels (A) prior to SHIV challenge in all animals and (B) during the challenges before transmission. Note that three control macaques and one LB-mCV-N macaque became infected at the first challenge and thus were excluded from this analysis.

**Table 1:** Pro-inflammatory cytokine levels in CVL (pg/ml).

A. Colonized and control animals, prior to SHIV challenges										
LB-CV-N colonized animals										
	IL-1β	IL-6	TNFα	IL-13	GM-CSF	IFNγ	IL-8	IL-12 p40	IL-17	IL-18
***min***	<0.07	<0.03	<0.5	<0.01	<0.03	<0.06	111	0.34	<0.02	1.0
***max***	243.9	917.8	25.8	2.0	8.5	1.8	10706	13.5	0.6	499
***median***	0.7	3.5	0.3	0.0	0.4	0.3	360	2.0	0.0	10.1
***n***	12	12	12	12	12	12	12	12	12	12
**Non-colonized control animals**										
***min***	<0.07	0.56	<0.5	<0.01	<0.03	<0.06	43.8	0.3	<0.02	0.9
***max***	148.8	48.6	11.3	1.5	2.9	1.0	2572	61.3	1.4	173
***median***	1.1	8.7	2.1	0.2	0.3	0.2	298	2.6	0.1	15.0
***n***	8	8	8	8	8	8	8	8	8	8
**B. Colonized and control animals during SHIV challenges, preinfection**										
**LB-CV-N colonized animals**										
	**IL-1β**	**IL-6**	**TNFα**	**IL-13**	**GM-CSF**	**IFNγ**	**IL-8**	**IL-12 p40**	**IL-17**	**IL-18**
***min***	0.8	2.1	<0.5	<0.01	<0.03	<0.06	489	0.9	<0.02	2.9
***max***	140	587	32.6	0.7	3.5	0.8	6368	7.0	0.3	104
***median***	2.9	36.9	0.6	0.0	0.6	0.2	1187	2.3	0.1	28.9
***n***	11	11	11	11	11	11	11	11	11	11
**Non-colonized control animals**										
***min***	1.6	3.7	<0.5	<0.01	<0.03	<0.06	160.0	1.0	<0.02	0.6
***max***	23.4	66.8	7.1	1.8	3.2	1.7	1235	7.5	0.6	138
***median***	3.0	8.6	0.4	0.4	0.1	0.4	347	1.9	0.1	11.9
***n***	5	5	5	5	5	5	5	5	5	5

**Figure 4:**
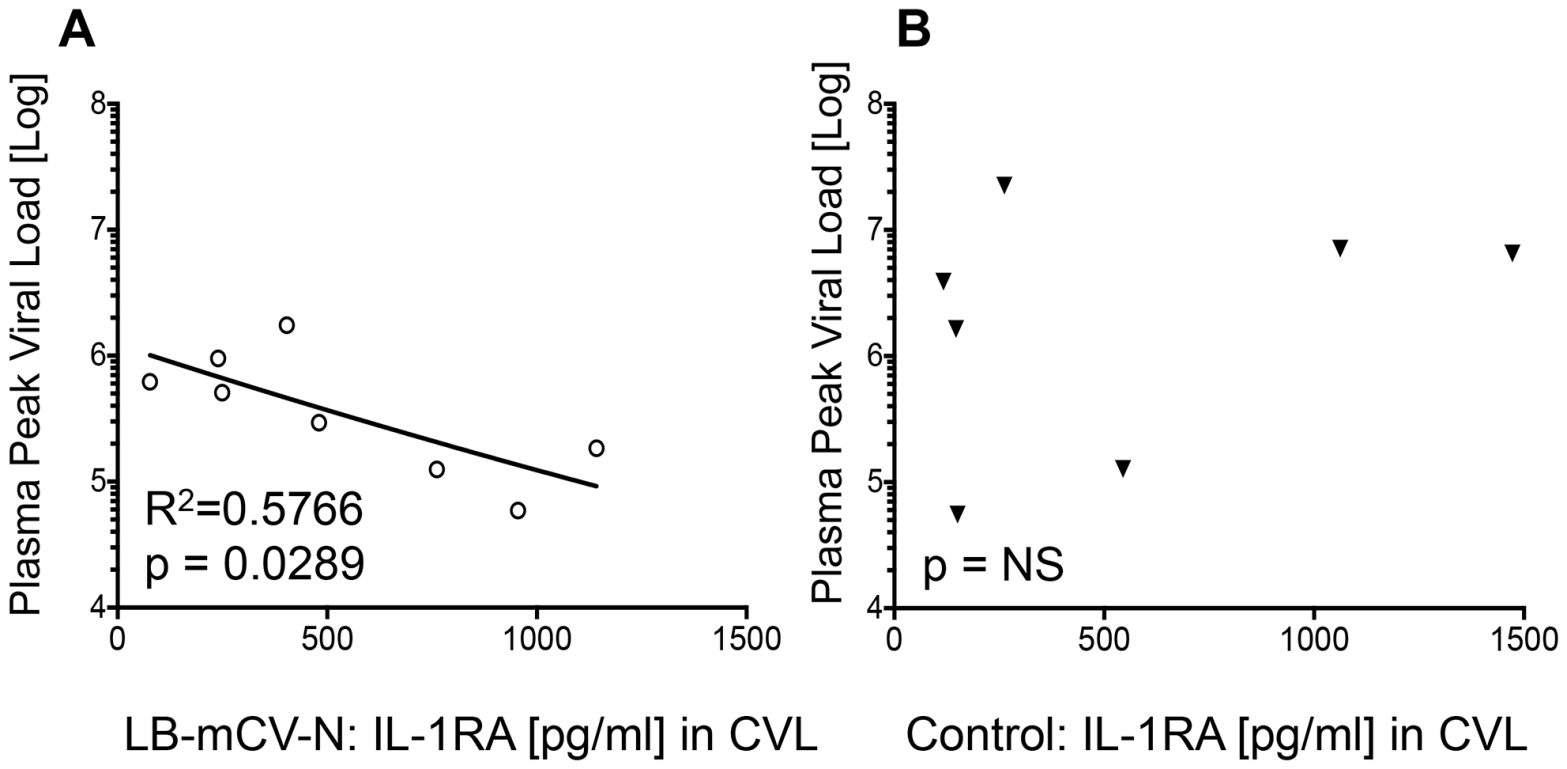
Higher mucosal IL-1RA was predictive of lower peak viral load. Macaques that were colonized with LB-mCV-N and experienced breakthrough infection had 6-fold lower viral loads than control macaques [29]. Mucosal IL-1RA was measured during SHIV challenge prior to infection and the average value was plotted against the peak viral load. We found that higher levels of mucosal IL-1RA prior to infection were correlated with a lower peak viral load. A) Levels of mucosal IL-1RA [pg/ml], found in CVLs of macaques colonized with LB-mCV-N, were inversely correlated with peak plasma viral load, thus higher IL-1RA prior to infection correlated with lower viral load B) No correlation with peak plasma viral load was found in mucosal IL-1RA, found in CVL of uncolonized control macaques.

During our challenge experiment, we reported that LB-mCV-N colonized macaques required more SHIV challenges to become infected (as shown in Figure 1) and LB-mCV-N colonized macaques with breakthrough infection had 6-fold lower viral load than control macaques [29]. We asked whether the elevated mucosal IL-1RA levels correlated with time to transmission or reduction in peak viral load. We found no correlation between IL-1RA levels and overall time to transmission. Interestingly mucosal IL-1RA levels in the CVL’s of macaques colonized with LB-mCV-N were inversely correlated with peak plasma viral load (Figure 4A). This correlation was not seen in the CVLs of control macaques (Figure 4B). Thus, during SHIV challenge, elevation in IL1-RA in CVL, associated with LB-mCV-N colonization, was also associated with lower peak viral load (R squared = 0.5766, p = 0.0289) in macaques with breakthrough infection.

## Discussion

One currently promising approach to the prevention of HIV transmission is the use of topical microbicides which, when applied on mucosal surfaces, block or inactivate the virus before it can reach a target cell. Irrespective of their mechanism and efficacy of viral inhibition, it is essential that such microbicides not alter the healthy microbiota or harm the mucosal environment in a way that could potentiate infection. The importance of careful safety testing in suitable animal models is highlighted by the unfortunate experience with two previously examined microbicides, N-9 and CS, both of which may have enhanced HIV acquisition in human clinical trials. Subsequent studies showed that these agents were associated with increased concentrations of pro-inflammatory cytokines in vaginal fluids [20], [23], [24] and may also have altered the vaginal microbiota, with decreases in naturally protective *Lactobacillus* [21]. The first successful human microbicide trial, CAPRISA-004, used 1% tenofovir gel, which does not have such harmful effects; however, the modest efficacy that was observed in this trial may be in part attributable to existing cervical-vaginal inflammation unrelated to the gel [16]. Thus, finding ways to reduce cervical-vaginal inflammation may critical to a comprehensive HIV prevention program.

We have recently described a successful test of a live microbicide using *Lactobacillus* as a delivery system for the HIV-1 entry inhibitor cyanovirin-N (LB-mCV-N), in a macaque model [29]. The purpose of this study was to determine the impact of vaginal colonization with the engineered LB-mCV-N on the vaginal environment, specifically on the vaginal microbiota, vaginal pH, and the extracellular components of innate immunity to determine safety.

Although we were able to achieve high numbers of LB-mCV-N in the macaque vaginal environment, the LB-mCV-N did not alter the overall species diversity or lower pH to the levels seen in women. Mirmonsef *et al*. reported that rhesus macaques (and pigtail macaques) have much lower levels of vaginal glycogen and lower lactic acid levels than humans [32]. Because glycogen serves as a carbon source for the production of lactic acid, the LB-mCV-N although capable of making high levels of lactic acid, [33] did not in this model, thus no concomitant (or only a modest) drop in pH occurred. In our study, cultures from the vaginas of control macaques contained mainly *Streptococcus, Staphylococcus,* and *Enterococcus species*, while three out of eight had endogenous *Lactobacillus sp*., thus the vaginal microbial composition of our experimental animals corresponds well to the previous reports [30], [32], [34]–[36].

Our live microbicide produces mCV-N constitutively on the cervical-vaginal mucosa and we found no evidence of local inflammation in this repeated challenge model [29]. A previous report suggested that CV-N (produced in *E. coli*) may not be safe for use as a topical microbicide, because when cultured with peripheral blood mononuclear cells (PBMCs) in an *in vitro* culture system the native CV-N protein activated the expression of multiple cytokines (IL-1β, IL-1RA, IL-2, IL-4, IL-5, IL-7, IL-8, IL-9, IL-10, IL-12, IL-14, IL-15, IL-17, Eotaxin, G-CSF, GM-CSF, IFN-γ, IP-10, MCP-1, MIP-1β, MIP-1α, PDGF, RANTES, TNFα, and VEGF [37]. In contrast to the *in vitro* PBMC culture system we found that *in vivo*, there was no evidence of immune activation by the mCV-N produced *in situ* by the LB-mCV-N bacteria. Indeed, the most noteworthy comparison was an apparent decrease in IL-13 (p = 0.0198*), in the CVLs of LB-mCV-N colonized macaques. In addition, with repeated dosing of LB-mCV-N during the challenge experiment we observed an increase in the levels of an anti-inflammatory cytokine IL-1RA (p = 0.013) over background, that was not observed with sequential measurements of cytokines in our first study [29]. We did not find significant elevation of any other cytokines that were measured. Thus, the PBMC *in vitro* system [37] was not predictive of what occurs *in vivo* on the mucosa, and may instead reflect endotoxin contamination present in the native CV-N sample produced in *E. coli*.

A potentially more useful system for testing microbicides was described by Fichorova *et al*. and involved the generation and characterization of several immortalized epithelial cell lines from human vagina, ectocervix and endocervix [38], [39]. These cell lines may be used to measure cytokines for the safety evaluation of topical microbicides, including live biotherapeutic products such as LB-mCV-N (*L. jensenii* 1153–1666) [40]. Yamamoto *et al*. reported that both LB-mCV-N, and the native/parental strain *L. jensenii* 1153 did not induce proinflammatory proteins, such as IL-8, TNF-α, and IL-6 [40]. Both *L. jensenii* 1153 and LB-mCV-N produced constant levels of IL-1RA when cultured with vaginal (Vk2/E6/E7) epithelium cells [40]. IL-1RA is believed to counteract the inflammatory effects of IL-1 proteins [41] and here we show an inverse correlation between median vaginal IL-1RA levels and subsequent peak viral load. This finding is consistent with results of *in vitro* experiments, which showed that IL-1RA blocked IL-1-mediated inductive effects on HIV virus production [42]. We speculate that the increased IL-1RA present in the mucosa at the time of SHIV infection in LB-mCV-N colonized macaques could have reduced inflammation leading to a smaller initial nidus of infection and subsequent modest reduction in peak viral load.

Our finding of decreased IL-13 is consistent with the increasing body of literature on *Lactobacillus* probiotic strains and thus is likely not associated with mCV-N protein produced *in situ* by LB-mCV-N, but instead by the *Lactobacillus* themselves. Decreases in IL-13 production was described as a property of various *Lactobacillus* strains, mainly in the connection with the suppression of allergic reactions and shift from TH2 towards TH1 type responses to a more balanced cytokine profile [43]–[47].

In these *in vivo* experiments mCV-N appeared to be safe, and did not trigger any proinflammatory changes to the local mucosal environment. A live microbicide approach that uses vaginal *Lactobacillus* as a delivery platform for proteins that inhibit HIV entry, may work in multiple ways; live microbicides may improve vaginal health in humans by reconstituting the vaginal microbiota, lowering pH and inflammation and delivering the anti-viral protein *in situ* at the site of HIV entry.

## Methods

### Ethics Statement

The twenty female Chinese origin rhesus macaques (Macaca mulatta), used in this study were housed at BIOQUAL, Inc., in accordance with the recommendations of the Association for Assessment and Accreditation of Laboratory Animal Care International Standards and with the recommendations in the Guide for the Care and Use of Laboratory Animals of the United States – National Institutes of Health. The Institutional Animal Use and Care Committee of BIOQUAL approved these experiments (study #3516). When immobilization was necessary, the animals were sedated by intramuscularly injection with 10 mg/kg of ketamine HCl and 1 mg/kg acepromazine. All efforts were made to minimize suffering. Details of animal welfare and steps taken to ameliorate suffering were in accordance with the recommendations of the Weatherall report, “The use of non-human primates in research”. Animals were housed in an air-conditioned facility with an ambient temperature of 21–25°C, a relative humidity of 40%–60% and a 12 h light/dark cycle. Animals were socially housed when possible or individually housed if no compatible pairing could be found. The animals were housed in suspended stainless steel wire-bottomed cages and provided with a commercial primate diet and fresh fruit twice daily, with water freely available at all times.All NHPs housed at BIOQUAL, Inc. receive bi-monthly behavioral assessments from one of two fulltime behaviorists. All NHPs receive at least three commercially available pet toys in their cages at all times to manipulate as part of their enriched environment. Choosing from a wide array of toys, each animal is provided one hard toy (i.e. hard plastic), one soft toy (i.e. soft rubber), and a third toy (i.e. hard plastic or soft rubber) chosen by the caretaker assigned to each animal. NHPs are given destructible enrichment as a way for them to alter their environment, release aggression/tension, and forage through. The destructible enrichment provided includes items such as cardboard, shredded paper, and phonebooks. Soft toys, such as fleece blankets and soft pet toys, are available for comfort to all NHPs. Many foraging opportunities are made available to all NHPs. Treats used for foraging and in feeders include, but are not limited to: Bunny Blocks, Nutra Blocks, Crumble Disks, PRIMA Treats, Fruity Gems, Fleece Foraging Crumbles, wax moth larvae (all available through BIOSERV), raisins, popcorn, nuts, birdseed, oatmeal, hardboiled eggs, species-specific food items, etc. All animals observed to exhibit any abnormal behavior(s) deemed detrimental to the physical and/or mental health of the individual are prescribed specific enrichment by the behavioral staff in order to decrease/eliminate/redirect such behaviors.

Following the final SHIV challenge, macaques were held for four additional weeks to monitor viral infection and viral set point. The viral set point was designed as the time point for euthanasia to avoid pain or suffering associated with SHIV clinical sequelae. Euthanasia was performed by IV injection of a lethal dose of pentobartbital (Beuthanasia-D) and cardiac arrest was confirmed by auscultation (in agreement with the 2007 AVMA Guidelines for Euthanasia).

### Study Animals

The colonization and SHIV_SF162P3_ challenge study has been previously described [29], four control macaques from the original study were not included in this analysis because insufficient pre-treatment samples were available. Swab samples for microbiological determination were collected first, followed by cervical-vaginal lavage (CVL), typically on a Monday. Animals were re-colonized with LB-mCV-N delivered in 3% hydroxyethylcellulose (HEC) gel for two days after sample collection, (Monday and Tuesday), SHIV challenge occurred on Wednesday at least 24 hrs after re-colonization. These additional doses were intended to standardize the levels of LB-mCV-N among animals, and also to replenish the LB-mCV-N that was removed during CVL and swab sampling. Except for week one, the culturable microbiota shown in Figure 1, represents a sample taken six days after colonization. Control macaques received only 3% HEC gel on the same schedule as the experimental group.

### Microbiology

The vaginal microbiota of each animal was sampled each week using the Port-A-Cul™ anaerobic collection and transport system (Becton Dickinson, Cockeysville, MD, USA). Swabs were collected and transported within 8 hrs. The swabs were plated using a four quadrant plating technique for isolation of aerobic bacteria on tryptic soy agar with 5% sheep blood agar, phenylethyl alcohol agar (PEA) with 5% sheep blood, MacConkey agar, and MRS agar (Becton Dickinson, Franklin Lakes, NJ). For isolation of anaerobic organisms the samples were plated to Brucella blood agar, PEA agar with 5% sheep blood, laked blood with kanamycin and vancomycin agar, and bacteroides bile esculin agar (Anaerobe Systems Morgan Hill, CA). Cultures on the primary plates were screened, and sub-cultured for isolation of pure cultures. Gram stain and API bacterial identification test strips (Biomerieux, St Louis, MO) were used for preliminary identification of the bacterial isolates. Aero-tolerance determination was used for primary facultative anaerobic bacterial identification, followed by Gram stain and API 20A identification kit. Bacterial identification to the species level was performed using 16S rRNA gene sequencing as previously described [30].

Semi -quantitative measurements of the microbiota were made on the growth in each quadrant of the plate, based on standards containing serial dilutions of *Lactobacillus jensenii* 1153–1666 plated with the same quadrant plating technique. Growth in quadrant 4 represented at least 10^6^ CFU, quadrant 3, 10^5^ CFU, quadrant 2, 10^4^ CFU, and quadrant 1, 10^3^ CFU. Stacked bar graphs are used to represent the proportional numbers of organisms found in each culture and semi-quantitative counts. Each species identified is labeled and color-coded in Figure 1. A Shannon diversity index was performed using SAS Statistical software.

### Vaginal pH

Vaginal pH was monitored using a portable HI 99181 Skin pH Meter Hanna Instrument® (Woonsocket, RI).

### Menstruation Evaluation

Menses was monitored by inserting a clean saline dampened cotton-tipped applicator into the monkey’s vagina. The swab was then removed and a recording from 0 to 3 was made (0 = no blood, 1 = slight, 2 = moderate, 3 = heavy bleeding). The monkeys were trained for this procedure by giving food as a reward. Late luteal phase (occurring prior to menses) has been reported to be associated with increased risk of vaginal SHIV infection [31].

#### Cytokine and immunoglobulin measurements from Cervical Vaginal Lavage (CVL)

CVLs were taken by instilling 2 mL of PBS spiked with complete, EDTA-free protease Inhibitor (Roche Applied Science, Indianapolis, IN) into the vagina, flushing it back and forth four times, and then collecting the fluids on ice. Samples were clarified by centrifugation at 820×g at +4°C and snap frozen on dry ice, prior to storage at −80°C until analysis. The levels of 23 cytokines in CVLs were determined from 20 of the macaques used in the challenge experiment, with a multiplexed fluorescent microsphere immunoassay using the Luminex 100 system (Luminex Corporation, Austin, TX) using Milliplex™ MAP Non-human primate cytokine kit (Millipore). Biochemical markers and cytokines that were measured include Granulocyte Colony Stimulating Factor (G-CSF), Granulocyte-Macrophage Colony Stimulating Factor GM-CSF, Interferon gamma (IFN-γ), Interleukin 1 beta (IL-1β), Interleukin 1 Receptor Antagonist (IL-1RA), Interleukin 2 (IL-2), Interleukin 4 (IL-4*), Interleukin 5 (IL-5), Interleukin 6 (IL-6), Interleukin 8 (IL-8), Interleukin 10 (IL-10), Interleukin 12/23 (IL12/23(p-40), Interleukin 13 (IL-13), Interleukin 15 (IL-15), Interleukin 17 (IL-17), Monocyte Chemoattractant Protein -1 (MCP-1), Macrophage Inflammatory Protein 1 beta (MIP-1β), Macrophage Inflammatory Protein 1 alpha (MIP-1α), soluble CD 40 Cluster of Differentiation Ligand (sCD40L), Transforming Growth Factor alpha (TGFα), Tumor Necrosis Factor alpha (TNFα), Vascular Endothelial Growth Factor (VEGF), and Interleukin 18 (IL-18). *(IL-4 was not detected or below the level of detection). Data were collected and analyzed with Bioplex Manager v3.0 software (Bio-Rad, Hercules, CA) using a 5-parameter fitting algorithm. To account for possible differences between tests, samples were analyzed in overlapping batches testing all the time points from the individual animals at the same time. All p values of 0.05 or less have been reported, and have not been corrected for multiple comparisons. Because of multiple (23) measurements, a cytokine/biomarker that reaches a value 0.05 may not be significant if a Bonferroni correction is applied.

### Statistical Analysis

The Statistics and Data Management Section of National Institutes of Heath performed statistical analyses on the data.

## Supporting Information

File S1 **Prevention of vaginal SHIV transmission in macaques by a live recombinant** ***Lactobacillus*** **.**
(PDF)
